# Calcareous sponges can synthesize their skeleton under short-term ocean acidification

**DOI:** 10.1038/s41598-023-33611-3

**Published:** 2023-04-25

**Authors:** Bárbara Ribeiro, Carolina Lima, Sara Emilly Pereira, Raquel Peixoto, Michelle Klautau

**Affiliations:** 1 TaxoN Laboratory, Zoology Department, Biology Institute, Federal University of Rio de Janeiro, Rio de Janeiro, 21941-599 Brazil; 2 Biological and Environmental Science and Engineering Division, Red Sea Research Center, King Abdullah University of Science and Technology, Thuwal, 23955-6900 Saudi Arabia

**Keywords:** Zoology, Ocean sciences

## Abstract

Calcifying organisms are considered as threatened by ocean acidification, because of their calcium carbonate skeleton. This study investigated if a calcareous sponge could synthesize its skeleton (i.e. spicules) under ocean-acidification conditions. Sponge cell aggregates that have the potential to develop into a functional sponge, called primmorphs, were submitted to a 5-day experiment, with two treatments: control (pH 8.1) and acidified conditions (pH 7.6). Primmorphs of the calcareous sponge *Paraleucilla magna* were able to synthesize a skeleton, even under low pH, and to develop into functional sponges. The spicules had the same shape in both conditions, although the spicules synthesized in low pH were slightly thinner than those in the control. These results suggest that *P*. *magna* may be able to survive near-future ocean-acidification conditions.

## Introduction

The ocean is undeniably warming due to human activities related to high emissions of greenhouse gases, especially carbon dioxide (CO_2_)^1^. In addition to ocean warming, ocean acidification (OA) is another significant threat to be addressed, as the ocean is the major sink of CO_2_. Carbon dioxide reacts with seawater to form carbonic acid (H_2_CO_3_), which dissociates into bicarbonate (HCO_3_^−^) and carbonate (CO_3_^−2^) by releasing hydrogen ions (H^+^). These changes in the carbonate chemistry of the ocean lower the pH and the availability of CO_3_^−2^ ions, which are essential for the formation of calcium carbonate (CaCO_3_), characterizing OA^2^. In this sense, it is often assumed that calcifying organisms are threatened by OA due to their composition of CaCO_3_ (e.g.^3,4^). However, a variety of effects, from negative to positive, have been observed in calcifying organisms in response to OA. According to a meta-analysis by Leung et al.^5^, the majority of the marine calcifiers (> 65%) show a neutral response regarding calcification and growth under near-future OA conditions (pH 7.90–7.61). Nevertheless, the carbonated substrates they built, such as reef frames, are still threatened (e.g.^6,7^). In addition, results obtained using specific organisms, in laboratory conditions, do not reflect ecological consequences of OA, such as community shifts from carbonated to fleshy algal dominated systems and biodiversity loss^8^. On the other hand, considering only the individuals, calcifying organisms in general would not be as threatened to OA as previously thought. This may also be the case for calcareous sponges, the only class of the phylum Porifera with CaCO_3_ spicules.

Smith et al.^9^ warned about the possible negative effects of OA on calcareous sponges due to the high-magnesium calcite composition of their skeletons, as this mineral phase of CaCO_3_ is the most soluble^10,11^. Nonetheless, only three studies have addressed the effects of OA on these animals^12–14^. Peck et al.^12^ observed an increase in the abundance of the calcareous sponge *Leucosolenia* sp. under OA conditions, suggesting a positive response of this species to this stressor (pH 7.7). The calcareous sponge *Leucetta chagosensis* showed tissue necrosis associated with warming, but no effects regarding acidification (pH 7.6)^13^. However, neither of these studies analyzed the skeleton of calcareous sponges under OA conditions. In the first report committed to fill this gap, Ribeiro et al.^14^ observed that spicules of adult individuals of the calcareous sponge *Sycettusa hastifera* were smaller (in both length and width) under low-pH conditions (pH 7.6), suggesting possible future problems regarding body support and/or water flux. Nevertheless, as only adult sponges were analyzed, the question remained of whether young sponges, which would be forming their entire skeleton and not only renovating spicules, could be more sensitive to OA. To answer this question, it is of utmost importance to observe the skeleton development in early life stages of calcareous sponges.

Hence, we performed a short-term experiment using primmorphs, sponge cell aggregates capable of forming functional sponges^15^, of the calcareous sponge *Paraleucilla magna*, aiming to verify the shape and size of the spicules in entirely synthesized skeletons under acidified conditions.

## Results

**Figure 1:**
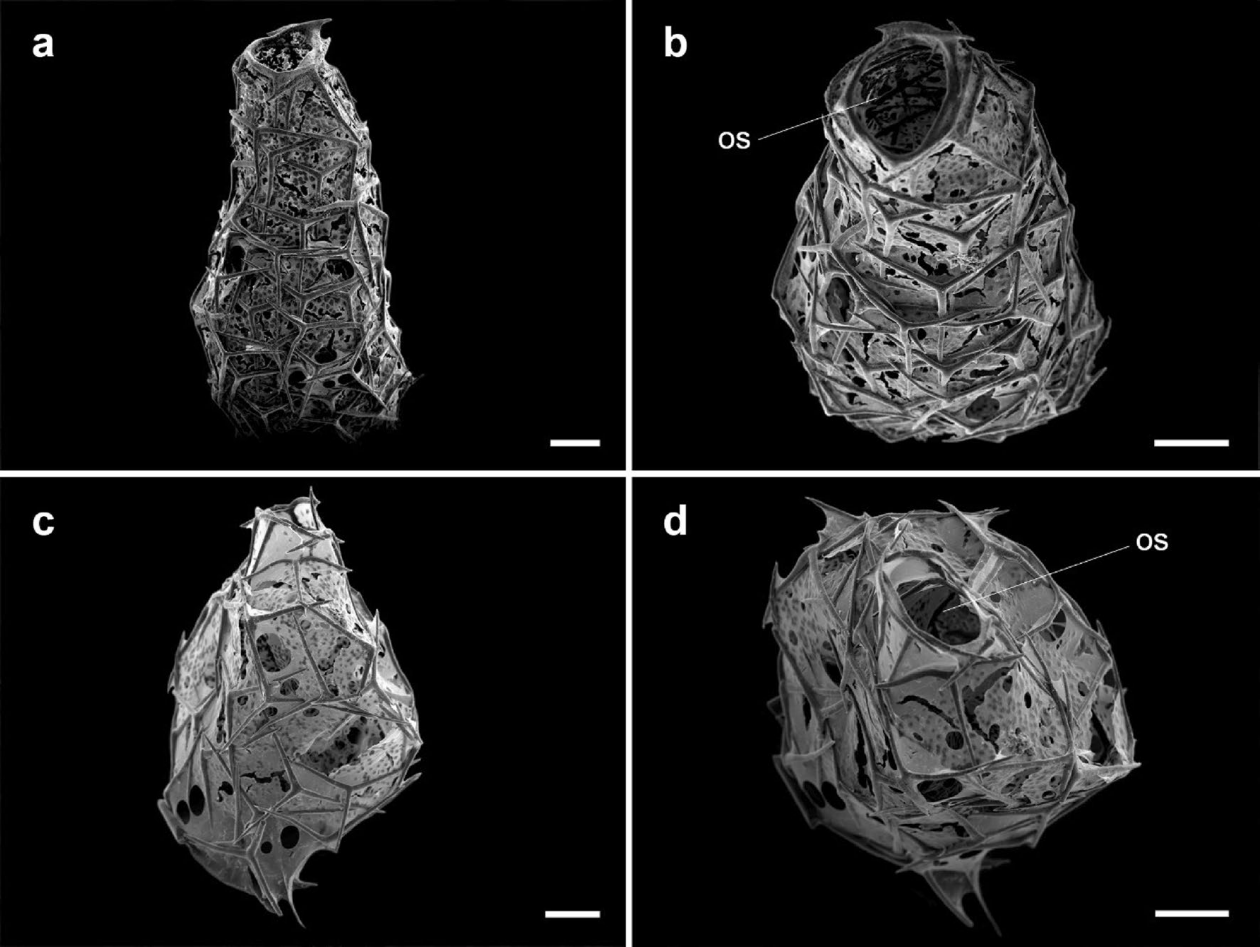
Sponges developed from the primmorphs of the calcareous sponge *Paraleucilla magna* in the experimental system, SEM images. (**a**, **b**) control; (**c**, **d**) low pH. Scale bars = 50 µm. *os* osculum.

At the end of the experiment, the primmorphs of *P*. *magna* had developed into functional sponges, with skeleton and aquiferous system, in both control and low-pH conditions (Fig. 1). A total of 49 young sponges were analyzed, 22 from the control and 27 from the low-pH treatments.

**Figure 2:**
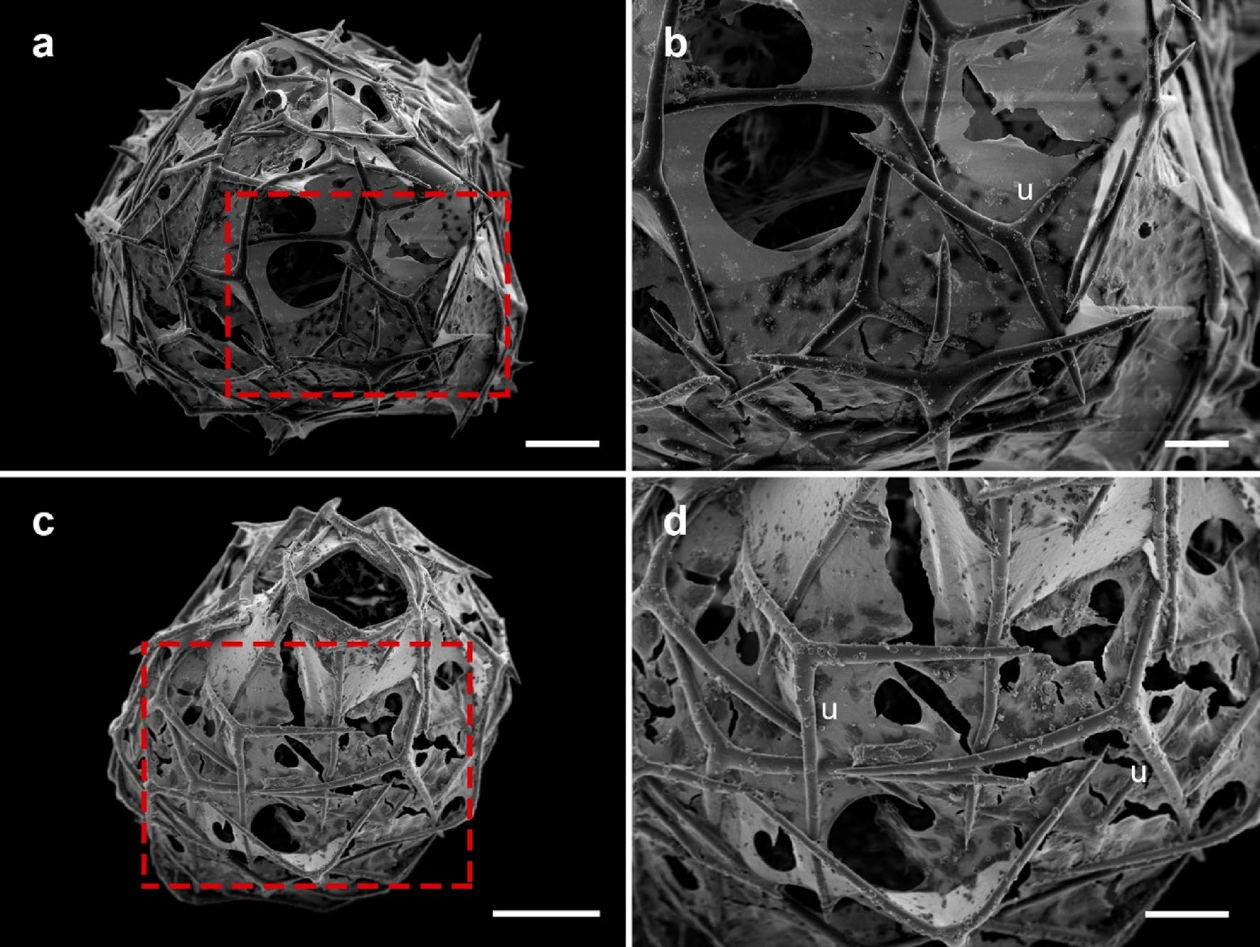
Detail of the shape of the spicules (SEM images) in (**a**, **b**) control; and (**c**, **d**) low pH. Red squares in (**a**) and (**c**) correspond to images (**b**) and (**d**), respectively. Scale bars = (**a**, **c**) 50 µm; and (**b**, **d**) 20 µm. u = unpaired actine.

**Figure 3:**
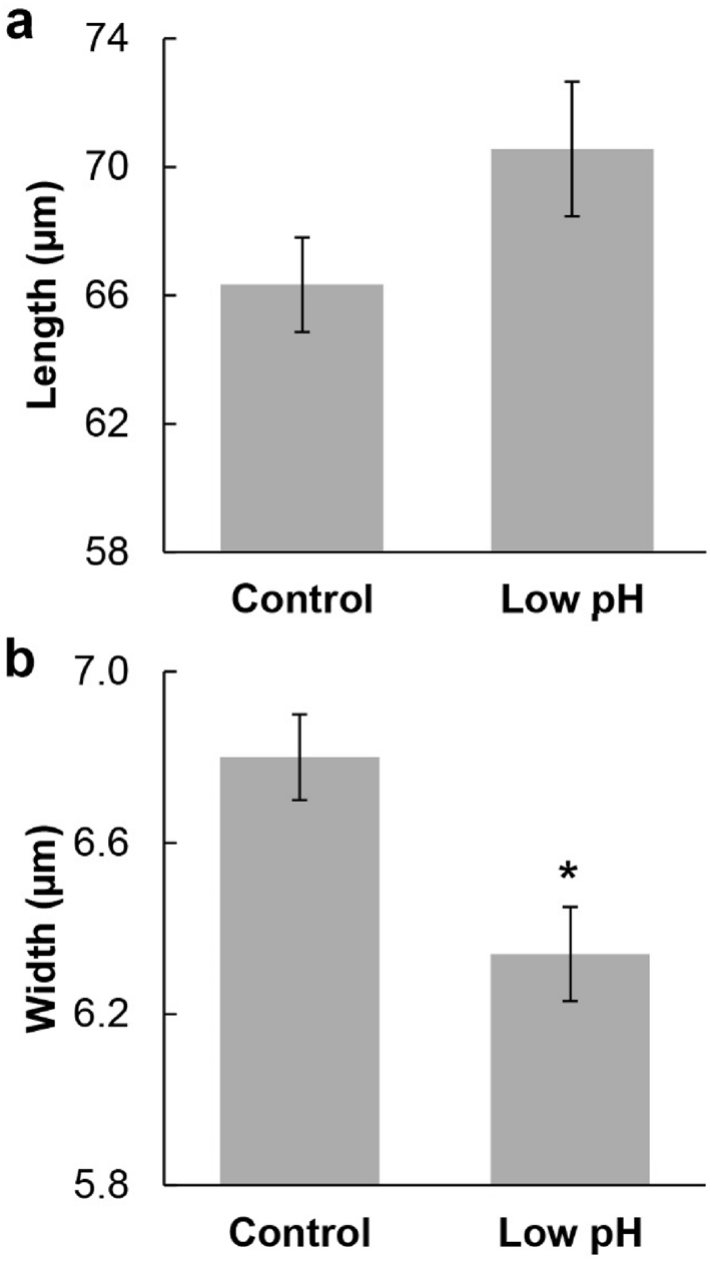
Mean measurements of the unpaired actines of the spicules synthesized during the ocean acidification experiment. (**a**) Length (N = 88 measurements per treatment); and (**b**) Width (N = 110 measurements per treatment). (*) thinner spicules under low-pH treatment (*p* = 0.002). Error bars represent the standard error.

In the newly formed sponges, the spicules had the same shape in both the control and low-pH conditions (Fig. 2). As it was not possible to distinguish between triactines and tetractines, we considered them only as cortical spicules. The unpaired actines did not differ in length between the control and low-pH treatment (K–W test, χ^2^ = 1.284, *p* = 0.257; Fig. 3a). However, the spicules were approximately 7% thinner under low pH (ANOVA, *F* = 9.666, *p* = 0.002; Fig. 3b).

## Discussion

Early life stages of calcifying marine organisms tend to be more sensitive than adults to the effects of near-future OA (pH 7.90–7.61)^5^. For example, larvae of the sea urchin *Sterechinus neumayeri* were shorter and malformed under acidified conditions (pH 7.8 and 7.6)^16^. Foster et al.^17^ observed severe deformities in the skeleton of juvenile coral *Acropora spicifera* growing under OA conditions (pH 7.7). In contrast, our results indicated low vulnerability of *P*. *magna* in an early life stage, as the sponges developed virtually the same in the control and acidified conditions (pH 7.6). Furthermore, we used the most severe OA prediction for the end of the twenty-first century, which reinforces the low vulnerability of *P*. *magna*. Only one study has reported OA impacts on early life stages of sponges, showing no deleterious effects of OA on the larvae and juveniles of the horny sponge *Phyllospongia foliascens*, which has a skeleton composed solely of spongin^18^. It is important to highlight that OA will likely impact sponge communities in different ways as responses are species-specific^19^.

The spicules of *P*. *magna* showed the same shape in the control and low-pH conditions, the same as in the skeleton of adult individuals^20^. This might suggest that the proteins involved in the synthesis of these spicules (see^21,22^) are not affected by OA. However, these proteins might be affected by temperature, as deformed spicules have been observed in *S*. *hastifera* under high temperatures^14^.

In acidified conditions, the spicules of *P*. *magna* were slightly thinner, but not shorter, whereas in *S*. *hastifera* the outermost spicules (diactines and cortical triactines) were both thinner and shorter^14^. This could be explained by the lower calcite saturation state (Ω) in low pH, as the calcification process can be impaired by lower ΩCaCO_3_ (see^23^). Nonetheless, the calcareous sponges were still able to synthesize their skeleton. Some marine calcifying organisms have mechanisms to cope with this lower ΩCaCO_3_ availability, such as the ability to control the pH at the calcification site regardless of the external pH (e.g.^23–26^). One of these pH-controlling mechanisms is related to the conversion of HCO_3_^−^ into CO_3_^−2^ via H^+^ regulation (e.g.^23^), and calcareous sponges may possess a similar mechanism. Coral-boring sponges, for example, are capable of decreasing the pH at the etching site in order to dissolve the CaCO_3_ by releasing H^+^ vesicles^27^.

Formation of thinner calcareous structures under near-future OA has been observed for some marine invertebrates, such as corals (e.g.^28^), molluscs (e.g.^29^), polychaetes (e.g.^30^), and sea urchins (e.g.^31,32^). In some cases, a thinner skeleton was also more fragile. For example, in the serpulid tubeworm *Hydroides elegans*, the tubes were thinner and broke more easily in low pH (pH 7.8)^30^. In the mussel *Mytilus californianus*, the shells secreted under near-future OA conditions (pH 7.75) were weaker, thinner, and smaller than in present-day seawater conditions^29^. Nevertheless, a weaker skeleton might not necessarily be associated with structural thinness, but rather with changes in the crystal morphology and/or chemical composition of Mg/Ca^29^. For this reason, in the case of calcareous sponges, it is also necessary to investigate the fracture toughness of these spicules, along with the biomineral structure and composition.

Here we demonstrated that the calcareous sponge *P*. *magna* can produce functional sponges from dissociated cells even under low pH. As primmorphs can start synthesizing spicules approximately from the sixth day after cell dissociation^33^, and the maintenance of these cells and young sponges in aquaria systems is quite challenging, we had to perform a short-term experiment. Then, our results may show some insights of what could happen in a future OA scenario for *P*. *magna*, as it was observed the synthesis of the entire skeleton. Investigation of OA effects (and any other anthropic stressor) in early life stages is crucial to predict the future of marine ecosystems (e.g.^34,35^), as these stages are usually more sensitive to stressors than adults^5^. Deleterious effects on early life stages have a cascade-effect in the environment, undermining the continuity of sponge populations. This study showed for the first time that calcareous sponges are capable of synthesizing a skeleton with a normal shape, although with slightly thinner spicules under OA. Although we can, at present, suggest that the calcareous sponge *P*. *magna* may be classified as a species with low vulnerability under near-future acidified conditions, investigation of the fracture toughness, biomineral structure, and chemical composition of its spicules are also needed, as a thinner and possible more fragile skeleton could lead to deleterious biological and/or ecological impacts.

## Methods

### Primmorphs preparation

One adult individual of the calcareous sponge *P*. *magna* was collected by snorkeling at Praia Vermelha, Rio de Janeiro, Brazil (22° 57′ 15.1″ S, 43° 09′ 50.5″ W) and transferred to the laboratory at the Federal University of Rio de Janeiro in a 0.5-L plastic bottle containing seawater at ambient temperature for 30 min.

**Figure 4:**
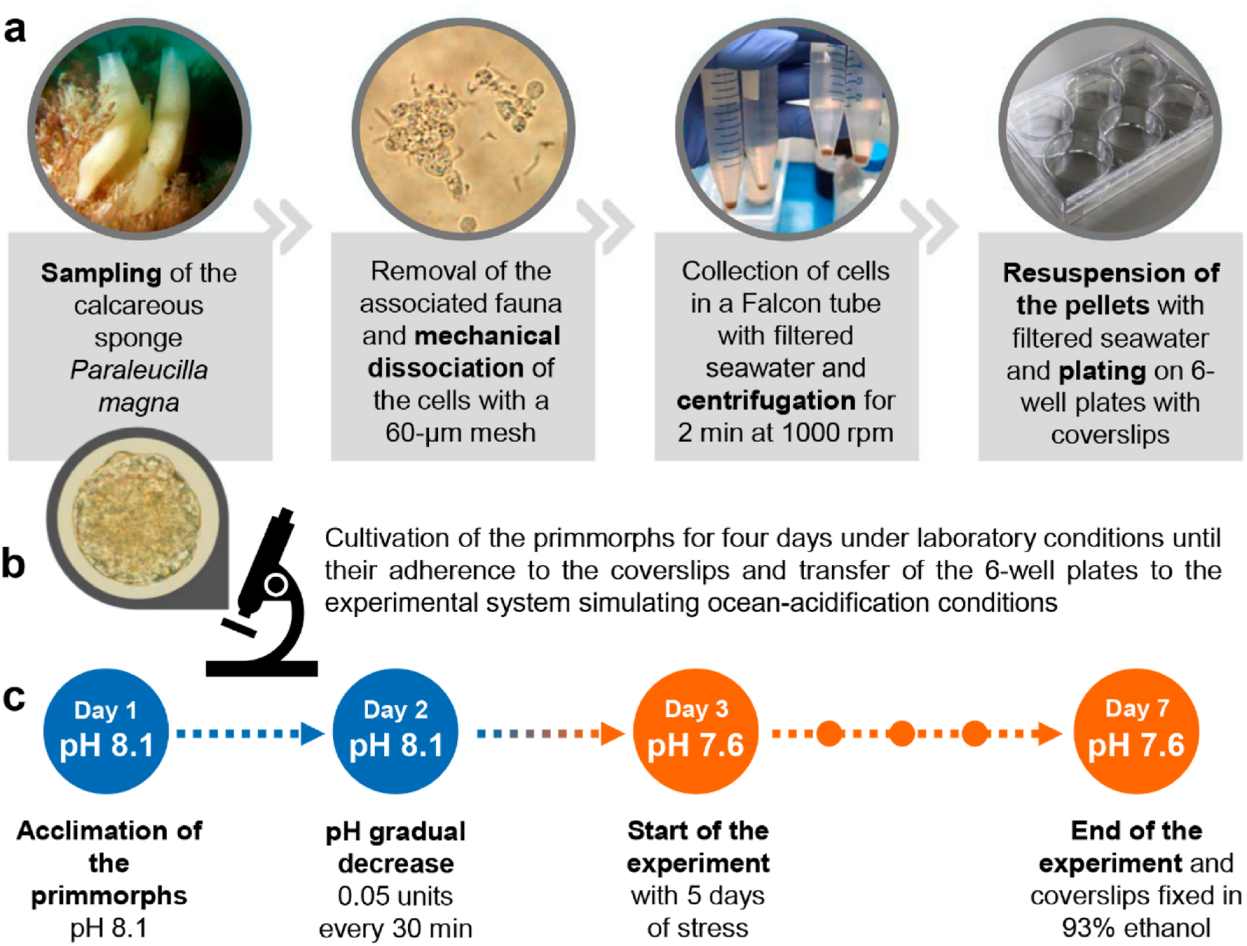
Experiment overview. (**a**) Preparation of primmorphs; (**b**) Cultivation of primmorphs in the laboratory; and (**c**) Experimental design of the ocean acidification experiment, using the calcareous sponge *Paraleucilla magna* as a model. Figure drawn with PowerPoint (Microsoft Office 365) and Canva Pro.

At the laboratory, the sponge was observed under a stereomicroscope and the associated fauna was carefully removed. Then, the sponge was squeezed through a 60-µm mesh for mechanical dissociation and its cells were collected in a 15-mL Falcon tube with 0.22 µm-filtered seawater. Cells were centrifuged for 2 min at 1000 rpm, the pellets were resuspended with filtered seawater, and plated in three wells of each of six 6-well plates containing coverslips (24 × 24 mm) and approximately 2 mL of filtered seawater (Fig. 4a). The plates were kept at room temperature and observed under an inverted microscope (Nikon). One-third of the filtered seawater was changed daily for four days, until the primmorphs adhered to the coverslips (Fig. 4b; Supplementary Fig. S1a).

### Experimental system

An open-system tank setup was constructed at the Rio de Janeiro Marine Aquarium Research Centre (AquaRio) to perform the experiment simulating ocean acidification (Supplementary Fig. S1b). The system comprised six aquaria of 8 L each (kept in a water bath to maintain the temperature at 24 °C), with open water flow, exchanging their volume five times a day. Each aquarium received one of the 6-well plates, which was kept open and weighted with fish weights to keep it at the bottom of the aquarium (Supplementary Fig. S1b).

Two treatments were performed, with three replicates each: control (pH 8.1) and low pH (pH 7.6). pH 7.6 was selected based on the worst-case scenario predicted by the Intergovernmental Panel on Climate Change (IPCC), which considers a decrease of 0.3–0.5 pH units by the end of the twenty-first century (RCP8.5)^36^. The experiment lasted seven days, with the first day (24 h) for acclimation, the second day to gradually reduce the pH, and 5 days of stress (Fig. 4c). The pH was reduced by approximately 0.05 units every 30 min until reaching the target pH of 7.6 (Supplementary Fig. S2), as similarly applied in other studies on calcareous sponges^13,14^. Each aquarium replica of the low-pH treatment had a separate system of CO_2_ injection, regulated by a solenoid valve linked to a pH controller (Analytical Instruments Model PH-803; probe precision 0.01 pH units). All aquaria were fitted with pumps (Sarlobetter Mini-A) to aid in water oxygenation, and the temperature was maintained at 24 °C with a chiller coupled to a water bath (Mundo Sub MS 3000). Temperature (Incoterm 5964), salinity (Hand-Held Analog Refractometer), pH (MS Tecnopon mPA 210; probe precision 0.01 pH units), and alkalinity (Gran method; Camourze-Mod, 1994) were measured daily. The other parameters of the carbonate system (*p*CO_2_ and Ωcalcite) were calculated indirectly, using the software CO_2_calc^37^ (Table 1; Supplementary Table S1).

**Table 1:** Summary of seawater chemistry parameters measured (*) and calculated (**) for the ocean acidification experiment, represented as the mean and standard deviation (between brackets) of measurements taken daily (for 5 days) for the three experimental replicates.

	Temperature (°C)*	pH (total)*	Salinity*	Alkalinity (µmol kg^−1^)*	*p*CO_2_ (µatm)**	Ω calcite**
Control	24 (2)	8.11 (0.05)	36 (1)	2359 (22)	327 (47)	5.92 (0.58)
Low pH	24 (2)	7.56 (0.07)	36 (1)	2351 (32)	1430 (260)	2.06 (0.34)

### Image processing and statistical analysis

At the end of the experiment, the coverslips were carefully removed from the 6-well plates and immediately fixed with 93% ethanol. The coverslips were placed on a stub, dried in a stove at 37 °C for approximately one hour before being sputter-coated with gold (UNIMICRO, UFRJ), and images were recorded under a scanning electron microscope (SEM; Jeol 6510) at the UFRJ Institute of Biology. The unpaired actines of the cortical spicules of the sponges were measured (length × width) from the SEM micrographs, using the software AxioVision 4.8.2 (Zeiss). In total, 88 measurements per treatment were made for the length of the spicules, and 110 measurements per treatment for the width (Supplementary Table S2). We were careful to measure only those actines that appeared entirely in the micrographs.

The normality and homogeneity of the measurement data were tested based on the Shapiro–Wilk and Bartlett tests, respectively, in order to perform the analysis of variance (ANOVA). When the data did not meet the assumptions of normality and homogeneity, the non-parametric Kruskal–Wallis (K–W) analysis was used. The statistical analyses were performed in the software R version 4.1.3 for Windows.

## Supplementary Information

Supplementary Information.

## Data Availability

The datasets generated during and/or analysed during the current study are available in Zenodo repository, https://doi.org/10.5281/zenodo.7534829.

## References

[1] Intergovernmental Panel on Climate Change (IPCC) (2021). Climate Change 2021: The Physical Science Basis.

[2] Gattuso JP, Hansson L, Gattuso JP, Hansson L (2011). Ocean acidification. Ocean Acidification: Background and History.

[3] Kleypas JA (1999). Geochemical consequences of increased atmospheric carbon dioxide on coral reefs. Science.

[4] Figuerola B (2021). A review and meta-analysis of potential impacts of ocean acidification on marine calcifiers from the Southern Ocean. Front. Mar. Sci..

[5] Leung JY, Zhang S, Connell SD (2022). Is ocean acidification really a threat to marine calcifiers? A systematic review and meta-analysis of 980+ studies spanning two decades. Small.

[6] Andersson AJ, Gledhill D (2013). Ocean acidification and coral reefs: Effects on breakdown, dissolution, and net ecosystem calcification. Ann. Rev. Mar. Sci..

[7] Schönberg CH, Fang JK, Carreiro-Silva M, Tribollet A, Wisshak M (2017). Bioerosion: The other ocean acidification problem. ICES J. Mar. Sci..

[8] Agostini S (2018). Ocean acidification drives community shifts towards simplified non-calcified habitats in a subtropical−temperate transition zone. Sci. Rep..

[9] Smith AM, Berman J, Key MM, Winter DJ (2013). Not all sponges will thrive in a high-CO_2_ ocean: Review of the mineralogy of calcifying sponges. Palaeogeogr. Palaeoclimatol. Palaeoecol..

[10] Andersson AJ, Mackenzie FT, Bates NR (2008). Life on the margin: Implications of ocean acidification on Mg-calcite, high latitude and cold-water marine calcifiers. Mar. Ecol. Prog. Ser..

[11] Haese RR, Smith J, Weber R, Trafford J (2014). High-magnesium calcite dissolution in tropical continental shelf sediments controlled by ocean acidification. Environ. Sci. Technol..

[12] Peck LS (2015). Acidification effects on biofouling communities: Winners and losers. Glob. Change Biol..

[13] Posadas N, Baquiran JIP, Nada MAL, Kelly M, Conaco C (2022). Microbiome diversity and host immune functions influence survivorship of sponge holobionts under future ocean conditions. ISME J..

[14] Ribeiro B (2021). Assessing skeleton and microbiome responses of a calcareous sponge under thermal and pH stresses. ICES J. Mar. Sci..

[15] Custodio MR (1998). Primmorphs generated from dissociated cells of the sponge *Suberites domuncula*: A model system for studies of cell proliferation and cell death. Mech. Ageing Dev..

[16] Byrne M (2013). Vulnerability of the calcifying larval stage of the Antarctic sea urchin *Sterechinus neumayeri* to near-future ocean acidification and warming. Glob. Change Biol..

[17] Foster T, Falter JL, McCulloch MT, Clode PL (2016). Ocean acidification causes structural deformities in juvenile coral skeletons. Sci. Adv..

[18] Bennett HM (2017). Interactive effects of temperature and pCO_2_ on sponges: From the cradle to the grave. Glob. Change Biol..

[19] Goodwin C, Rodolfo-Metalpa R, Picton B, Hall-Spencer JM (2014). Effects of ocean acidification on sponge communities. Mar. Ecol..

[20] Klautau M, Monteiro L, Borojevic R (2004). First occurrence of the genus *Paraleucilla* (Calcarea, Porifera) in the Atlantic Ocean: *P. magna* sp. nov.. Zootaxa.

[21] Voigt O, Adamski M, Sluzek K, Adamska M (2014). Calcareous sponge genomes reveal complex evolution of α-carbonic anhydrases and two key biomineralization enzymes. BMC Evol. Biol..

[22] Voigt O (2021). Carbonic anhydrases: An ancient tool in calcareous sponge biomineralization. Front. Genet..

[23] Ries JB, Cohen AL, McCorkle DC (2009). Marine calcifiers exhibit mixed responses to CO_2_-induced ocean acidification. Geology.

[24] Cameron JN (1985). Post-moult calcification in the blue crab (*Callinectes sapidus*): Relationships between apparent net H^+^ excretion, calcium and bicarbonate. J. Exp. Biol..

[25] de Nooijer LJ, Toyofuku T, Kitazato H (2009). Foraminifera promote calcification by elevating their intracellular pH. Pro. Nat. Acad. Sci..

[26] Cornwall CE (2018). Resistance of corals and coralline algae to ocean acidification: Physiological control of calcification under natural pH variability. Pro. Royal Soc. B..

[27] Webb AE, Pomponi SA, van Duyl FC, Reichart GJ, de Nooijer LJ (2019). pH regulation and tissue coordination pathways promote calcium carbonate bioerosion by excavating sponges. Sci. Rep..

[28] Tambutté E (2015). Morphological plasticity of the coral skeleton under CO_2_-driven seawater acidification. Nat. Commun..

[29] Gaylord B (2011). Functional impacts of ocean acidification in an ecologically critical foundation species. J. Exp. Biol..

[30] Li C (2014). Weakening mechanisms of the serpulid tube in a high-CO_2_ world. Environ. Sci. Technol..

[31] Byrne M (2014). Warming influences Mg^2+^ content, while warming and acidification influence calcification and test strength of a sea urchin. Environ. Sci. Technol..

[32] Rodríguez A, Hernández JC, Brito A, Clemente S (2017). Effects of ocean acidification on juveniles sea urchins: Predator-prey interactions. J. Exp. Mar. Biol. Ecol..

[33] Lanna E, Klautau M (2019). The choanoderm of *Sycettusa hastifera* (Calcarea, Porifera) is able to generate new individuals. Invertebr. Biol..

[34] Albright R (2011). Reviewing the effects of ocean acidification on sexual reproduction and early life history stages of reef-building corals. J. Mar. Biol..

[35] Przeslawski R, Byrne M, Mellin C (2015). A review and meta-analysis of the effects of multiple abiotic stressors on marine embryos and larvae. Glob. Change Biol..

[36] Intergovernmental Panel on Climate Change (IPCC). *Climate Change 2014: Synthesis Report* (Geneva, Switzerland, 2014).

[37] Robbins LL, Hansen ME, Kleypas JA, Meylan SC (2010). CO_2_calc: A user-friendly seawater carbon calculator for Windows, Mac OS X, and iOS (iPhone). U.S. Geol. Surv. Open-File Rep..

