# Supplementary material for: Calcareous sponges can synthesize their skeleton under short-term ocean acidification
Source: Sci Rep. 2023 Apr 25;13:6776. doi: 10.1038/s41598-023-33611-3 (PMC10130156; doi:10.1038/s41598-023-33611-3)
Supplement: Supplementary file 1 — Supplementary Information. [file 41598_2023_33611_MOESM1_ESM.docx]

**SUPPLEMENTARY DATA**

**Table S1:** Daily seawater chemical parameters measured (*) and calculated (**) for each aquarium replicate during stress. (a – c) control; (d – f) low pH. SD = standard deviation.

|  |  | **Temperature (°C)*** | **pH (total)*** | **Salinity*** | **Alkalinity (µmol kg^–1^)*** | ***p*CO_2_ (µatm)**** | **Ω calcite**** |
| --- | --- | --- | --- | --- | --- | --- | --- |
| a) | Day 3 | 21 | 8.01 | 38 | 2368 | 433 | 4.61 |
|  | Day 4 | 24.5 | 8.1 | 37 | 2367 | 335 | 5.93 |
|  | Day 5 | 24 | 8.13 | 37 | 2368 | 307 | 6.16 |
|  | Day 6 | 25 | 8.14 | 35 | 2343 | 300 | 6.27 |
|  | Day 7 | 25 | 8.15 | 34 | 2342 | 294 | 6.33 |
|  | **MEAN** | **24** | **8.11** | **36** | **2358** | **334** | **5.86** |
|  | **SD** | **2** | **0.06** | **2** | **14** | **57** | **0.72** |
| b) | Day 3 | 21 | 8.01 | 38 | 2367 | 433 | 4.61 |
|  | Day 4 | 24.5 | 8.1 | 37 | 2369 | 335 | 5.94 |
|  | Day 5 | 24 | 8.14 | 37 | 2368 | 298 | 6.26 |
|  | Day 6 | 25 | 8.12 | 35 | 2342 | 318 | 6.06 |
|  | Day 7 | 25 | 8.16 | 34 | 2319 | 283 | 6.37 |
|  | **MEAN** | **24** | **8.11** | **36** | **2353** | **333** | **5.85** |
|  | **SD** | **2** | **0.06** | **2** | **22** | **59** | **0.71** |
| c) | Day 3 | 21 | 8.15 | 38 | 2368 | 291 | 5.90 |
|  | Day 4 | 24.5 | 8.11 | 37 | 2390 | 328 | 6.10 |
|  | Day 5 | 24 | 8.12 | 37 | 2365 | 316 | 6.05 |
|  | Day 6 | 25 | 8.16 | 35 | 2389 | 289 | 6.62 |
|  | Day 7 | 25 | 8.08 | 35 | 2319 | 353 | 5.60 |
|  | **MEAN** | **24** | **8.12** | **36** | **2366** | **315** | **6.05** |
|  | **SD** | **2** | **0.03** | **1** | **29** | **27** | **0.37** |
| d) | Day 3 | 21 | 7.43 | 38 | 2365 | 1,938 | 1.427 |
|  | Day 4 | 24.5 | 7.61 | 37 | 2368 | 1,257 | 2.334 |
|  | Day 5 | 24 | 7.53 | 37 | 2368 | 1,536 | 1.941 |
|  | Day 6 | 25 | 7.62 | 36 | 2366 | 1,232 | 2.393 |
|  | Day 7 | 25 | 7.70 | 35 | 2292 | 978 | 2.697 |
|  | **MEAN** | **24** | **7.58** | **37** | **2352** | **1388** | **2.16** |
|  | **SD** | **2** | **0.10** | **1** | **33** | **365** | **0.49** |
| e) | Day 3 | 21 | 7.59 | 38 | 2368 | 1,305 | 2.008 |
|  | Day 4 | 24.5 | 7.53 | 37 | 2367 | 1,537 | 1.974 |
|  | Day 5 | 24 | 7.61 | 37 | 2346 | 1,244 | 2.273 |
|  | Day 6 | 25 | 7.58 | 36 | 2342 | 1,349 | 2.180 |
|  | Day 7 | 25 | 7.55 | 35 | 2292 | 1,431 | 1.979 |
|  | **MEAN** | **24** | **7.57** | **37** | **2343** | **1373** | **2.08** |
|  | **SD** | **2** | **0.03** | **1** | **31** | **114** | **0.14** |
| f) | Day 3 | 21 | 7.57 | 38 | 2368 | 1,372 | 1.926 |
|  | Day 4 | 24.5 | 7.64 | 37 | 2366 | 1,164 | 2.480 |
|  | Day 5 | 24 | 7.53 | 37 | 2392 | 1,552 | 1.961 |
|  | Day 6 | 25 | 7.48 | 35 | 2368 | 1,761 | 1.764 |
|  | Day 7 | 25 | 7.46 | 34 | 2291 | 1,799 | 1.613 |
|  | **MEAN** | **24** | **7.54** | **36** | **2357** | **1530** | **1.95** |
|  | **SD** | **2** | **0.07** | **2** | **38** | **267** | **0.33** |

**Table S2:** Measurements of the unpaired actine of *Paraleucilla magna*’s newly sponges developed under control and low-pH conditions. Minimum, mean, maximum, standard deviation (SD), standard error (SE), and N values for length and width of the unpaired actines of each treatment.

|  | **Length (µm)** | | | | | |  | **Width (µm)** | | | | | |
| --- | --- | --- | --- | --- | --- | --- | --- | --- | --- | --- | --- | --- | --- |
|  | Min | Mean | Max | SD | SE | N |  | Min | Mean | Max | SD | SE | N |
| **Control** | 35.21 | 66.33 | 93.35 | 13.77 | 1.47 | 88 |  | 3.99 | 6.80 | 9.87 | 1.03 | 0.10 | 110 |
| **Low pH** | 34.95 | 70.56 | 115.32 | 19.71 | 2.10 | 88 |  | 3.79 | 6.34 | 8.95 | 1.15 | 0.11 | 110 |

**
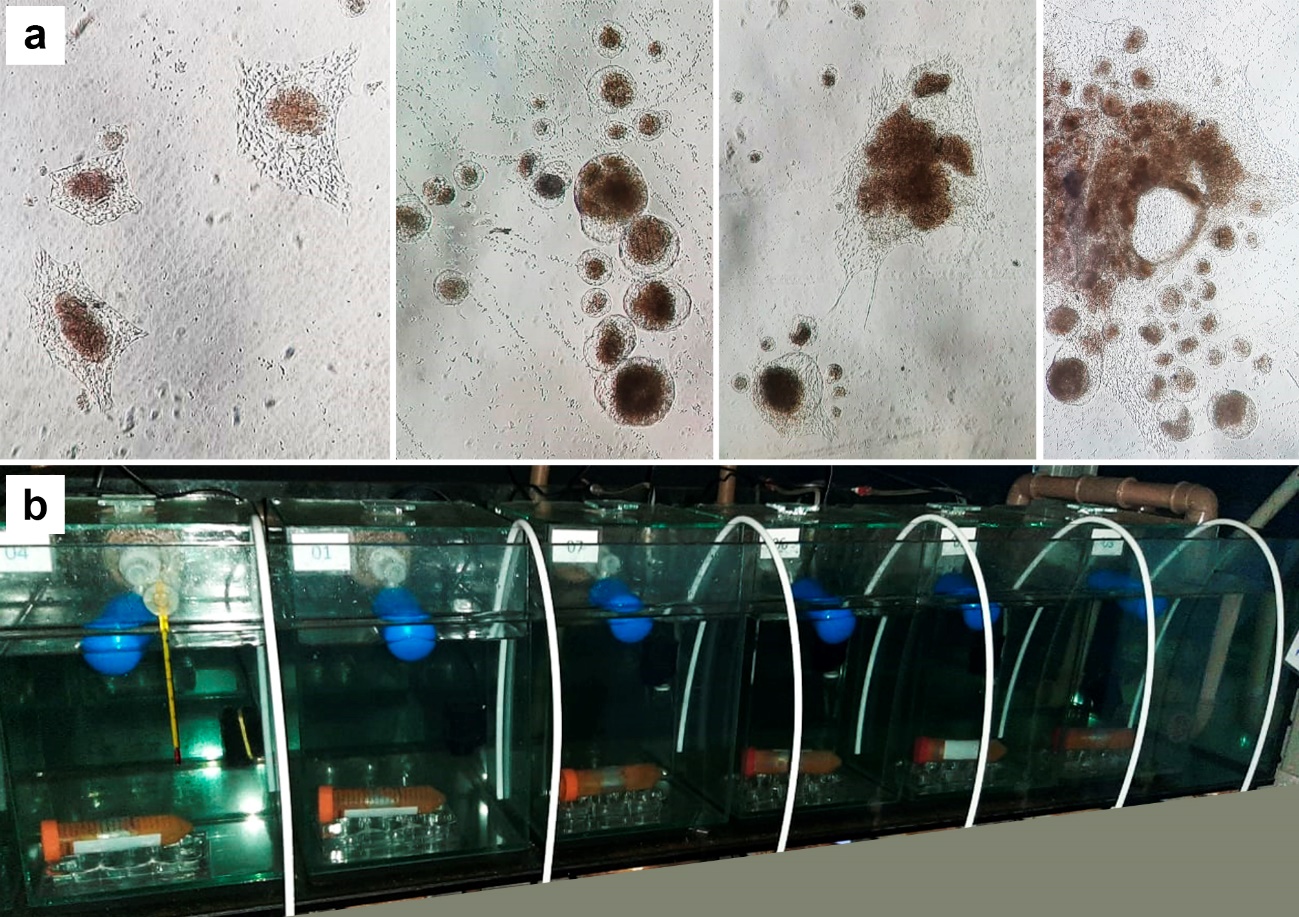
Figure S1:** (a) Primmorphs of the calcareous sponge *Paraleucilla magna* four days after cell plating and before the beginning of the (b) ocean acidification experiment (Photo: B. Ribeiro).


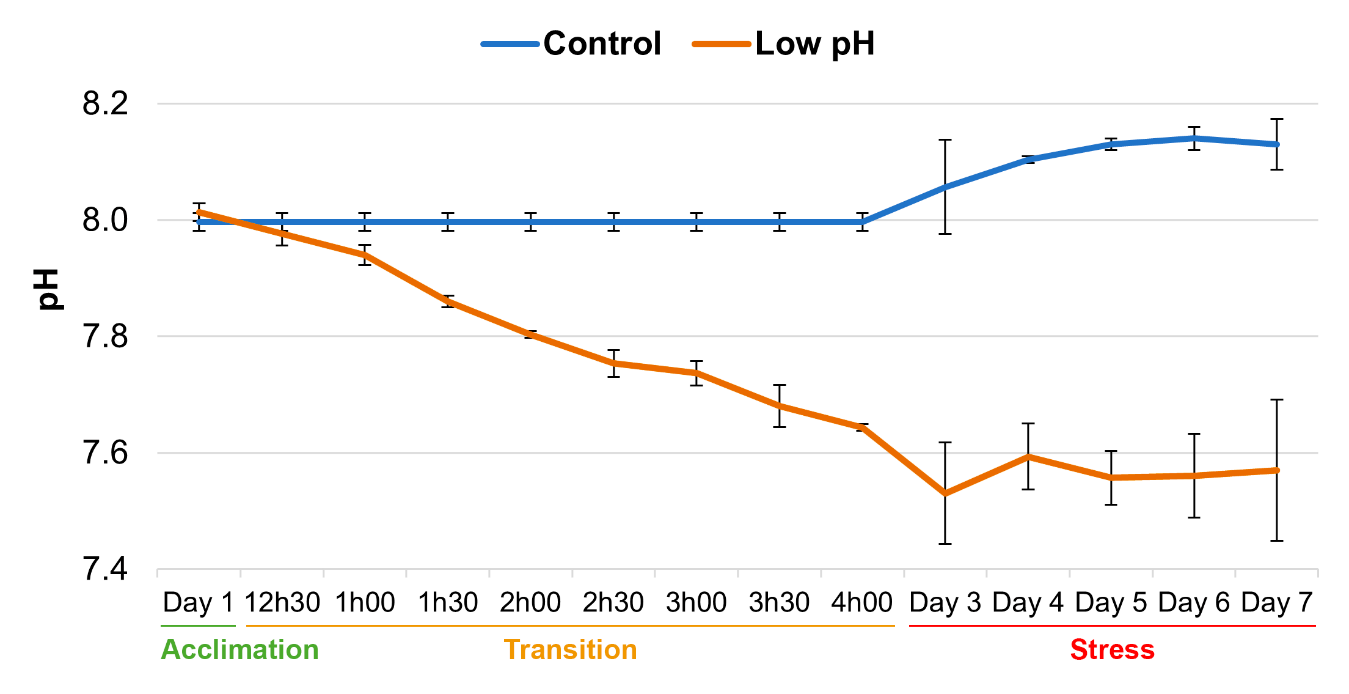


**Figure S2:** Daily pH measurements for the control and low-pH treatment. The transition to low pH was performed on day 2. Bars represent the standard deviation of the replicates.
